# Novel protocol for mapping virus integration sites in genes involved in therapy resistance

**DOI:** 10.1038/s41598-025-05160-4

**Published:** 2025-07-01

**Authors:** Michele Massimino, Zhangzan Huang, Jean Helmijr, Camilla Muritti, Anna Uijtdewillegen, Paolo Vigneri, John W. M. Martens, Maurice P. H. M. Jansen

**Affiliations:** 1 Department of Medical Oncology, Erasmus MC Cancer Institute, University Medical Centre Rotterdam, Dr. Molewaterplein 40, 3015 GD Rotterdam, The Netherlands; 2 Department of General Surgery and Medical-Surgical Specialties, University of Catania, Catania, Italy; 3 Department of Clinical and Experimental Medicine, University of Catania, Catania, Italy; 4 Center of Experimental Oncology and Hematology, A.O.U. Policlinico “G. Rodolico - S. Marco”, Catania, Italy; 5 Division of Oncology, Humanitas Istituto Clinico Catanese, 95045 Misterbianco, Catania Italy

**Keywords:** Virus integration sites, NGS, Endocrine resistant breast cancer, Breast cancer, Cancer models, PCR-based techniques, Next-generation sequencing, Molecular biology, Oncology

## Abstract

Retroviral transduction of cancer cell lines has been used to find genes related to therapy resistance. Characterization of virus integration sites (VIS) pinpointing these genes has been cumbersome. This study defines a sequencing-based protocol to rapidly characterize genomic loci near VIS. The protocol selectively amplifies VIS-genome junctions using the retroviral vector neomycin (NEO) gene, Genomic Walker Adapter approach, linker-mediated NEO-PCR (LM-NEO-PCR) or biotinylated NEO-capture, followed by long terminal repeats PCR (LTR-PCR). LTR-genome junctions were sequenced (NGS), reads mapped, quantified, and linked to genes. The protocol was tested on DNA from single clones holding twenty reported VIS loci and on multiplex and diluted clone DNA samples. Our VIS-NGS protocol enriched significantly (*p* < 0.02) more loci at high reads coverage in samples with VIS compared to negative controls. The protocol found seventeen reported VIS loci (85%) in single clone DNAs, of which fifteen loci (88%) were also detected in multiplex samples. Six loci were evaluated for all dilutions, with three loci detected at lowest 1% clone proportion. The protocol can be conducted in two weeks and successfully found almost all VIS loci in single VIS clones and detected half of the evaluated loci at low clone proportion.

## Introduction

Retroviruses integrate their genetic material into their host genome causing local modification of both genome architecture and gene expression of adjacent genes^1,2^. Altered gene expression is driven by the viral Long Terminal Repeat (LTR) regions which act as promoter regulatory elements for genes of the host near the junction between the LTR and the host, the so-called Virus Integration Sites (VISs)^1^. Diverse methods have been applied to characterize in detail drivers of disease and identify therapy resistance mechanisms. Historically, retroviral screens in cell lines. Other methods have been proposed to improve the VIS identification and analysis such as the CRISPR/Cas9-based approaches^3–5^. However, this needs a complex multiple-steps experimental procedure and, also, presents a strong limitation particularly concerning the gRNA design. Despite CRISPR/Cas9 editing system being a revolutionary molecular approach to genetically modify a cell to study in clinical research genetic disorders, gRNA can recognize off-target causing non-specific cleavage of genomic DNA^6–8^.

A retroviral screen to investigate endocrine therapy resistance in breast cancer was applied decades ago by our institute^9,10^. This screen was performed on the fully estrogen dependent breast cancer cell line ZR75.1, and cultured with tamoxifen, a commonly used endocrine treatment for estrogen receptor positive breast cancer. It resulted in tamoxifen resistant clones, which were picked and sub-cultured. Subcultures of these resistant clones were then characterized for their VIS loci, using restriction enzyme digestion and Sanger-sequencing. Individual VIS loci were confirmed to result in tamoxifen resistance after transfecting ZR75.1 and MCF7 breast cancer cells with gene specific cDNA expression vectors^11,12^. Expression of these VIS target genes associated also with progression-free survival in patients treated with tamoxifen^13,14^. The first identification and characterization of VIS loci in this retroviral screen at that time, however, was a cumbersome and prompt procedure, taking months of work.

VIS assays play a critical role in investigating the biological significance of the integrated retrovirus genome and different methods including those combined with NGS have been proposed. However, these methods show limitations including (i) restriction enzymes-based approach^15^, which can cause the failing of VIS identification. This especially occurs when only one enzyme is used and it is not near to virus-genome junction; (ii) used only for a specific virus type^16^ or (iii) requires the association between complex techniques, as Mu method^17^, followed by pyrosequencing which lack the sensitivity and specificity when compared with the next-generation sequencing approaches.

Lastly, linear-amplification mediated PCR (LAM-PCR)^18^, linker-mediated PCR (LM-PCR)^19^ and bidirectional retroviral integration site PCR^20^ methods have been developed. All methods showed high efficiency but present a significant restriction concerning their property to amplify both LTR regions resulting in two DNA products. One having the VIS and the second holding the undesirable internal virus sequence. Hence, if on one side, this event was requested by bidirectional-based approach, on the other hand it is unwanted by LAM- and LM-PCR methods and a restriction enzyme step was used to avoid the amplification of the internal virus sequence.

With the present work we provide a restriction enzyme-independent optimized VIS-NGS workflow to quickly find VIS loci on retrovirally transduced tamoxifen-resistant ZR75.1 breast cancer cell line clones to find genes involved in endocrine resistance mechanisms.

## Methods

**Fig. 1:**
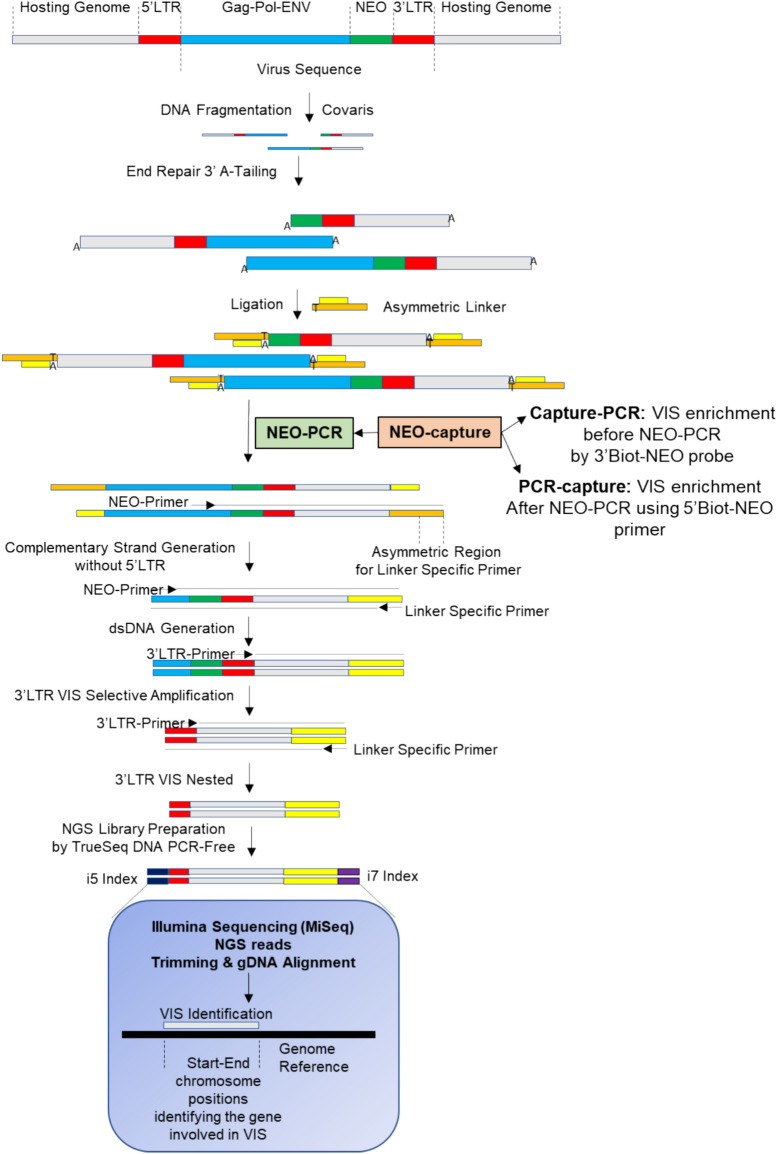
VIS-NGS workflow overview: LM-PCR, NEO-PCR, LTR-PCRs, Capture methods, and NGS library preparation. The different steps of the VIS-NGS protocol are illustrated as described in the materials & methods section.

The VIS-NGS protocol we describe here was designed to selectively amplify genomic regions having 3′-LTR sequences near to genes involved in the VIS, schematically shown in Fig. 1. We briefly describe important steps of the protocol, including experimental procedures and bioinformatic analyses for the identification of VIS loci. The “step-by-step” details of our approach can be found in the supplementary methods file. The protocol was evaluated on genomic DNA (gDNA) from ZR75.1 parental cells (negative controls) and on gDNA from four established tamoxifen resistant ZR75.1 VIS clones holding reported VIS loci.

Starting from fragmented gDNA, a “genome walker adapter system” was applied, followed by linker-mediated PCR (LM-PCR) to find unknown genomic regions next to a known genomic sequence. Samples have been enriched using a specific primer recognizing the neomycin virus sequence followed by 3′LTR PCR (LM-NEO-PCR). In addition, a capture protocol for the specific enrichment of VIS having gDNA was evaluated, using this VIS capture before and after neomycin PCR (NEO-PCR, Fig. 1). These were called capture-PCR (before NEO-PCR) and PCR-capture (after NEO-PCR), respectively. Hence, the obtained 3′LTR-VIS genomic regions were selectively amplified and used for NGS library generation and sequencing.

### Construct of genome walker adapter for viral integration libraries generation

A Genome Walker adapter system was used to find unknown sequences next to known genomic regions. The gDNA was sheared by a COVARIS ultra-sonicator to obtain ~ 1.000 bp DNA fragments, which were purified with AMPure XP beads (Beckman). Then Klenow exo-enzyme was used for blunt-end repair and 3′A-tailing of the sticky-end DNA breaks, followed by asymmetric T-linker ligation. Genome walker adapters were generated using asymmetric short and long oligonucleotide linkers having 3′dT-overhangs and 3′end-NH2 groups to prevent 3′end DNA polymerase extension and direct amplification toward the (genomic) region of interest.

### 3′LTR specific LM-NEO-PCR for VIS amplification

A NEO specific forward primer together with a linker specific reverse primer was used for LM-NEO-PCR to specifically amplify in 3′LTR direction containing the virus-genome junction. To improve virus sequence enrichment, LM-PCR was performed using a higher concentration of NEO-specific primer than linker-specific primer. Two rounds of PCR were performed to amplify the VIS genomic region using two 3′LTR forward primers and a linker specific reverse primer. Finally, a 3′LTR PCR was followed by a nested 3′LTR PCR.

### Capture before and after NEO-PCR of VIS holding gDNA

**Fig. 2:**
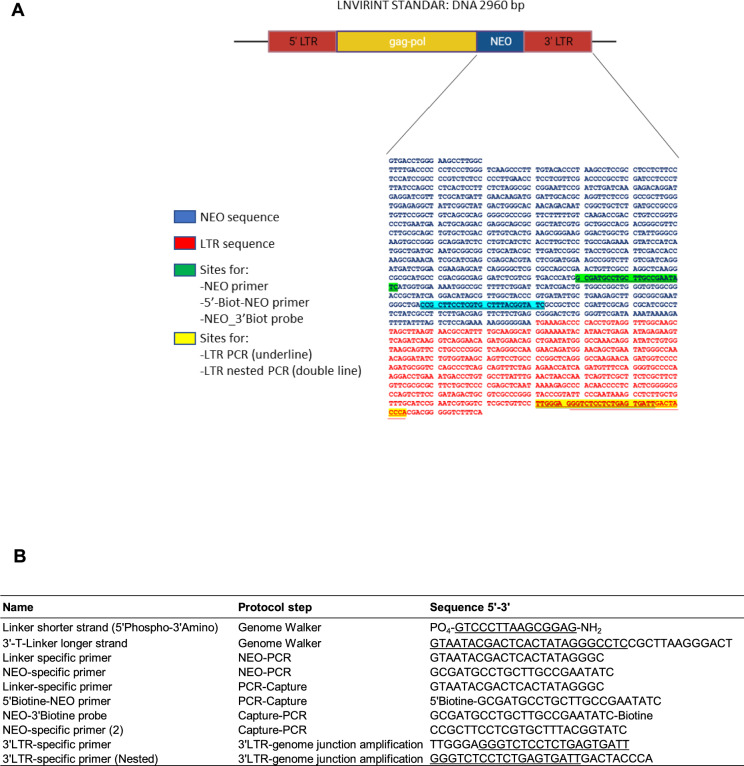
Virus sequence highlighting sites used for primers and probe design and details of linkers, primers and probes used in the protocol. Details of the retroviral vector (**A**) and primers and probes (**B**) used in the protocol.

Next, we also assessed a capture protocol for the specific enrichment of VIS holding gDNA and used this VIS capture before (capture-PCR) and after (PCR-capture) neomycin PCR (Fig. 1, Supplemental Figure S1). The capture was done by a biotinylated neomycin 5′end primer after NEO-PCR and biotinylated 3′end probe before NEO-PCR followed by Streptavidin beads incubation to remove gDNA without virus specific sequences and to enrich VIS holding gDNA. Figure 2 shows the virus sequence, primers and probe used in the described procedures, and details of linkers, primers and probes sequences (Supplemental Table S1).

### NGS library preparation, bioinformatics, and VIS locus detection

We used the TruSeq DNA PCR-free NGS library preparation protocol from Illumina to reduce PCR bias. This protocol included Nextera Illumina i5 and i7 dual-index ligation as adapters. The VIS-NGS libraries (2 nM) were sequenced on a Mi-Seq Illumina platform. The sequencing of VIS-libraries generated paired-end FASTQ files. The NGS data were trimmed and mapped using cutadapt (v3.7) and BWA (v07.17) tools, respectively. Next, SAMTOOLS (V1.9) aligned reads and determined coverage and mapping quality of reads. Reads within a 300 bp window were merged and considered as a single VIS, then linked to (neighboring) genes annotated by NCBI Refseq (hg38). Reads were normalized per million bases (RPMs) for comparison of samples and experimental setups, and several detection thresholds were explored to define the best settings for VIS locus characterization.

### Samples, experiments, and reported VIS loci

Our optimized LM-NEO-PCR and capture NGS-workflow protocols to find VIS loci were evaluated in different experimental setups using DNA samples from parental ZR75.1 cells and from tamoxifen resistant ZR75.1 VIS-clones VIII-18, VIII-20, VIII-24, and XII-7 (4). To verify the efficacy of the applied methods, the evaluation was focused on the twenty reported VIS loci from these VIS-clones (Table 1). The tamoxifen resistant VIS clones were evaluated as pure single clone DNA-sample (100% fraction) and as multiplex samples mixed in dilutions to obtain clones at fractions ranging from 25 to 1%. These latter multiplex samples were investigated to mimic the original retroviral transduced cell line having multiple VIS-clones at diverse cell number fractions.

**Table 1:** The VIS loci previously identified and reported as candidate target genes in the tamoxifen resistant clones VIII-18, VIII-20, VIII-24 and XII-7 from a retroviral screen in ZR75.1.

VIII-18	VIII-20	VIII-24	XII-7
*BNIP3L/PNMA2*	*LARP*	*FLJ12750/VPS37A/VPS37B*	*C9ORF86/RABL6*
*FLJ35036/ZBTB38*	*NARG2/ICE2*	*TRERF1*	*ASH1L*
*RAP2C*	*NEDD8*		*AGPS*
*TRPS1*			*EGFR*
*FSTL5*			*SNRPD1*
*SRD5AP1*			*FLJ20273/RBM47*
*LOC391679*			*LRP1B*
			*TUBA6/TUBA1C*

In total 33 specimens, containing none (negative controls), single up to four VIS clones, were evaluated (Supplemental Table S2). The LM-NEO-PCR method started with 30 or 25 PCR amplification cycles, the latter to limit false discovery due to VIS locus detection at low coverage (below 100RPM). In eight parental (negative control) specimens only 5 loci were detected, which were all not reported and almost all identified by capture methods (Supplemental Table S2). In the multiplex samples, two to four VIS clones were combined at different fractions, occasionally in the background of parental DNA. Supplemental Tables S2 and S3 summarize the characteristics of single and complex VIS clone samples and show the results of the NGS analyses from the applied methods for the published VIS loci. Reliable detection of three loci from the VIII-18 clone failed quality control and were excluded: Two VIS loci (*FSTL5*, *SRD5AP1*) were found only once and at low read coverage (< 100RPMs), while one locus (*LOC391679*) was not detected. Further analyses were focused on the remaining seventeen reported VIS loci (Supplemental Table S3).

### Confirmation of novel VIS loci by PCR and gel electrophoresis analysis

We designed new independent VIS locus-specific PCR assays for an additional validation of VIS loci (one reported VIS, six novel VIS) identified by VIS-NGS and evaluated the amplified PCR fragment sizes by gel electrophoresis. We used a universal virus-specific neomycin primer (NeoP: 5′- GCGATGCCTGCTTGCCGAATATC-3′) and designed VIS locus-specific forward and reverse primers (LSP) ~ 200 bp up- and downstream of the virus integration site. The neoP primer acts as forward primer when the VIS and locus are in sense orientation while it acts as reverse primer when the VIS and locus are in anti-sense orientation. Both forward and reverse LSPs were combined with the NeoP and tested, but only one LSP with the opposite orientation compared to neoP, resulted in > 1000 bp fragment PCR amplicons (FigureS 5D-E, Supplemental Figure S2). The VIS locus-specific primers (Supplemental Table S4) were designed for *BNIP3L* (an already reported VIS locus) as positive control, and for the novel VIS loci found by VIS-NGS in clones VIII-18 and -20 (*DPM3*, *MIR1908*, *KB1930G5.4*, *SNORA63*, *GLB1L* (clone VIII-18), and *MSL1* (clone VIII-20)). The PCR was performed on a Biorad T100 thermal cycler using a 50 µL PCR reaction mix containing 1X GoTaq Flexi Buffer (Promega, cat.no M890A), 2 mM MgCl2 (Promega, cat.no A351H), 0.2 mM dNTPs (NEB, cat. No N0447S), 1.0 µM neomycin-specific primer (NeoP), 1.0 µM locus-specific primer (LSP), 1.25 U GoTaq G2 Flexi DNA polymerase (Promega, cat.no M780B), 10% Glycerol (Sigma-Aldrich, cat. No. G5516), UltraPure™ Distilled Water (Invitrogen, cat. No 10977035) and 10 ng gDNA. The PCR cycling conditions are 1 cycle of 2 min initial denaturation at 95 °C, followed by 40 cycles of 10 sec denaturation at 95 °C, annealing for 30 sec at 55 °C, elongation for 4 min at 68 °C, and subsequently 1 cycle final elongation for 10 min at 68 °C, and cooling at 4 °C. The PCR was performed with the neoP combined with either the forward or with the reverse LSP in separate reactions on negative controls (No Template Control (NTC) and parental DNA (no VIS) and on DNA containing VIS derived from clones VIII-18, -20, and -24. The PCR fragment sizes were evaluated on a 1% agarose gel (Fisher Scientific, Catalog No. 16500–500), and samples were run at 180 V for 45 min. The samples loaded on the gel were 1.5 µL of Gene ruler 1 kb marker (Thermo Fisher Scientific, Catalog No. SM0311) and per VIS locus 10 µL sample containing two-thirds of PCR product and one-third of GelRed prestain loading buffer (VWR, Catalog No. BTIU41003). The agarose gels were visualized and recorded by the ChemiDoc system (Biorad ChemiDoc MP imaging system).

## Results

### Description of LM-NEO-PCR-based VIS-NGS protocol and study design

The goal of this study was to develop a rapid restriction-enzyme independent sequencing-based protocol for the characterization of virus integration sites. The VIS-NGS protocol used, based on LM-NEO-PCR, is shown in Fig. 1 and outlined in detail in the methods. It aims to enrich VIS loci specifically first by a primer recognizing the neomycin gene in the used recombinant retroviral vector introduced by insertional mutagenesis followed by a nested 3′LTR PCR together selectively amplifying genomic regions of interest having a 3′-LTR retroviral sequence in their locus. This protocol was performed with 25 and 30 PCR amplification cycles to compare their performance in VIS enrichment. Our designed LM-NEO-PCR was performed as a standalone protocol or to improve further VIS enrichment combined with a neomycin capture protocol with either a 3′-end biotinylated neomycin primer before (Capture-PCR) or a 5′-end biotinylated neomycin primer after LM-NEO-PCR (PCR-Capture) (Supplemental Figure S1). The enriched 3′LTR-VIS genomic junctions were then before or after capture amplified by nested LTR-PCR as in the standalone protocol and sequenced by NGS. The purpose of this latter experiment was to find the best method for specific and sensitive VIS containing locus enrichment to allow detection of VIS loci present at low frequency. The VIS-NGS findings are reported in the next paragraphs to assess the performance of our developed protocol.

To show that our method can find all VIS sequences present in a particular sample previously infected with a retrovirus and is suitable for screening purposes, we need ground truth and therefore we used DNA from four formerly generated and carefully characterized tamoxifen resistant subclones of the breast cancer cell line ZR75.1: subclones VIII-18, VIII-20, VIII-24, and XII-7 (4). These four subclones were previously traditionally characterized and carried respectively 7 (*BNIP3L/PNMA2, FLJ35036/ZBTB38, RAP2C, TRPS1, FSTL5, SRD5AP1, LOC391679),* 3 *(LARP, NARG2, NEDD8),* 2 *(FLJ12750, TRERF1)* and 8* (C9ORF86/RABL6, ASH1L, AGPS, EGFR, SNRPD1, FLJ20273/RBM47, LRP1B, TUBA6/TUBA1C*) VIS loci in their genomes (Table 1). The DNA samples from these pure clones carrying listed VIS loci were first evaluated by VIS-NGS using the LM-NEO-PCR protocol alone followed by NGS. Additionally, mentioned clones were diluted and mixed to better mimic multiplex samples from primary VIS-screens and to get an indication of the sensitivity and specificity of our method. Dilution series of 2 to 4 clones yielded multiplex samples with clones being present in proportions from 1% to 2.5%, 9%, 20% and 25%. These composed multiplex samples were evaluated with LM-NEO-PCR alone or using this method combined with one of the two neomycin capture methods (Capture-PCR, or PCR-Capture) again followed by NGS (Fig. 1, Supplemental Table S3). For each of the mentioned conditions after NGS on- and off-target VIS locus read coverage RPM was determined.

### Overall performance of the LM-NEO-PCR-based NGS protocol for VIS detection

**Fig. 3:**
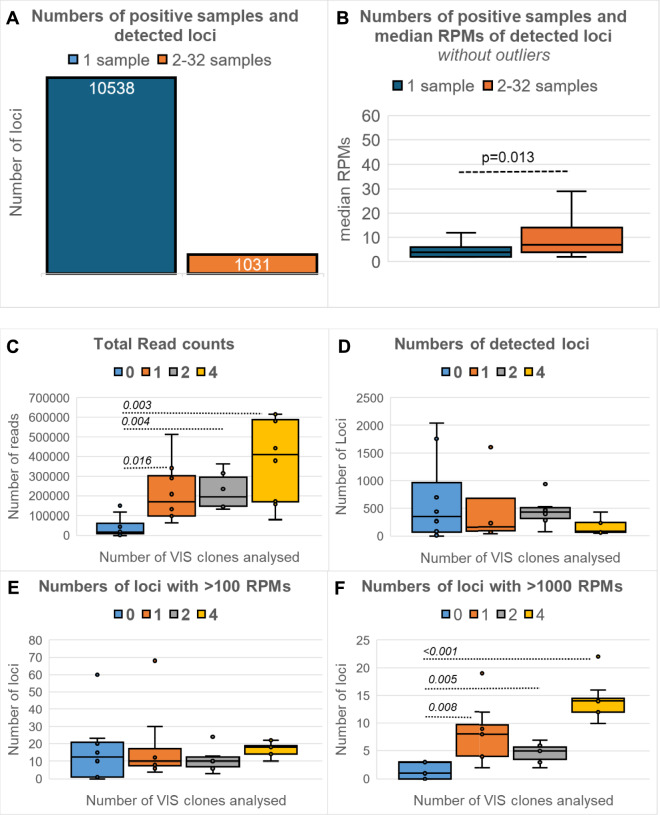
Overall performance of the VIS-NGS protocol. The VIS-NGS protocol with LM-NEO-PCR only or combined with NEO-capture followed by NGS detected 12,857 loci in 33 samples, including replicate samples. Most loci were detected in only one sample, while 1031 loci were identified in at least 2 samples (**A**). Loci in one sample were detected at low read coverage (reads per million bases (RPMs), presumably noise, whereas the loci identified in at least 2 samples were detected at higher read coverage (*p* = 0.013). Median read coverages are presented excluding outliers in both groups (**B**). Total read counts coverage and number of detected loci was compared between samples without VIS loci (negative controls, blue boxes), and samples with VIS loci from single pure clone (orange boxes), and from composed mixed and diluted samples having two clones (grey boxes) or four clones (yellow boxes). The VIS-NGS protocol produced significantly (*p* < 0.016) more reads in samples having VIS loci compared to the negative control samples (**C**). Horizontal dashed lines show comparison and their *p* values (student T-test). No significant (*p* > 0.12) differences were seen in the total number detected loci between samples with clones and samples without clones (negative control) (**D**). (**E**) and (**F**) show the number of loci detected when applying two thresholds on read coverage for VIS locus calling (loci with > 100 RPMs and with > 1000 RPMs) were compared between samples without clones (negative controls) and samples with clones. Only loci with > 1000 RPMs resulted in significant (*p* < 0.008) more loci in samples with clones compared to negative controls (**F**). No significant differences in number of loci with > 100 RPMs were seen between samples with and without clones (*p* > 0.09) (**E**). Horizontal dashed lines show comparison and their *p* values (student T-test).

**Fig. 4:**
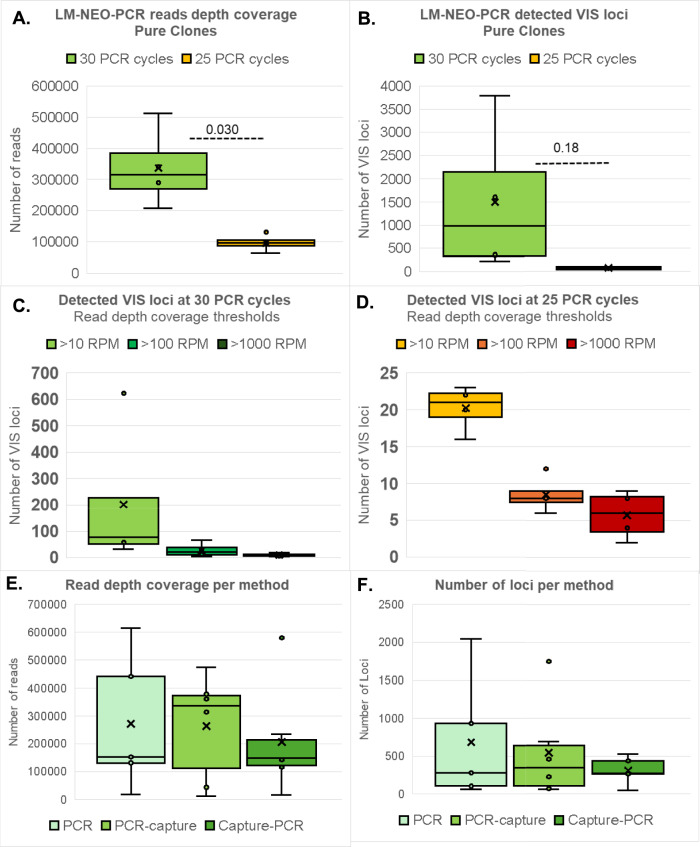
VIS-NGS performance at two different number of PCR amplification cycles and using LM-NEO-PCR alone or combined with NEO-capture. The VIS-NGS performance was evaluated for the two different number of PCR amplification cycles used in the protocol and for the three methods applied. (**A**)–(**D**) presents the NGS-findings obtained in single pure clone samples after 25 and 30 PCR amplification cycles. (**A**) and (**B**) show the overall reads coverage in reads per million bases (RPMs) (**A**) and number of VIS loci (**B**) after LM-NEO-PCR-based VIS-NGS analyses of single clone samples comparing 25 and 30 PCR amplification cycles. The protocol with 30 PCR cycles resulted as expected in significantly more RPMs for all loci compared to 25 PCR cycles (*p* = 0.030) The number of VIS loci after 30 PCR cycles was also higher than after 25 PCR cycles but not significantly different (*p* = 0.18). (**C**) and (**D**) shows the number of VIS loci called at three different thresholds for read depth coverage after 30 PCR cycles (**C**) and after 25 PCR cycles (**D**) No significant differences were observed in the number of VIS loci between 30 and 25 PCR cycles called at > 10 RPM (*p* = 0.29), > 100 RPM (*p* = 0.25), and > 1000 RPM (*p* = 0.23). (**E**) and (**F**) show the overall reads coverage in RPMs and number of VIS loci in composed mixed and diluted samples with 2 and 4 clones using LM-NEO-PCR alone or combined with NEO-capture before (capture-PCR) or after LM-NEO-PCR (PCR-capture). No significant differences were seen in read coverage (**E**) nor in number of VIS loci (**F**) between methods, however, LM-NEO-PCR alone and PCR-capture resulted in more RPMs than the capture-PCR method (**E**).

First, the protocol was analyzed for its overall performance in (replicate) DNA samples with and without VIS loci. Therefore, to notify overall differences all mentioned pure and composed multiplex samples were evaluated together independent from experimental setup and irrespective of the inclusion of an additional NEO-based capture step. In all samples with at least 2 reads detection threshold, a total of 11,569 genomic loci were detected with a median coverage of 4 RPMs (Range maximum reads: 2–353,326 RPMs). Most loci were detected in only one sample (n = 10,538) with a median coverage of 4 RPMs (Range 2–17,739 RPMs). In contract loci that were observed in at least two samples, 1031 in total, had significant (*p* = 0.013) higher reads coverage (median coverage = 7 RPMs; range 2–8306 RPMs) (Fig. 3A,B). In addition, samples without VIS loci (Parental DNA: negative controls, blue box) had significantly (*p* < 0.02) less total NGS reads compared to pure (orange box) or mixed samples (grey & yellow box) having VIS loci (Fig. 3C). The total number of detected loci (i.e. genomic regions having reads) was highest in the negative controls and samples with two clones and lowest in samples with four clones containing VIS loci (Fig. 3D). Significant differences were not seen between samples with one VIS clone all analyzed by LM-NEO-PCR alone and samples with two or four VIS clones which were in part evaluated by LM-NEO-PCR combined with NEO-capture (Fig. 3D). This analysis shows that our protocol is sensitive and not only finds VIS loci (on-target) but also irrelevant loci (off-target) like those detected in the negative controls, but these latter loci were detected mostly in one sample and had low coverage (< 100 RPMs). When arbitrarily selecting loci based on read coverage (Fig. 3E,F), loci with a high read coverage, i.e. more than 1000 RPM, were significantly more frequently present in samples with clones having VIS loci than negative controls (*p* < 0.008) (Fig. 3F). Additionally, a low and high number of PCR amplification cycles and LM-NEO-PCR alone or combined with capture methods were tested in the experiments (Supplemental Table S2) and compared for their performance. As expected, using 25 instead of 30 PCR amplification cycles in pure samples resulted in less reads coverage (Fig. 4A, *p* = 0.03) and lower number of VIS loci detected (Fig. 4B, *p* = 0.18). Only a minority of VIS loci were detected at > 100 and 1000RPMs (Fig. 4C,D) without significant differences between 25 and 30 PCR cycles (*p* = 0.25 and *p* = 0.23, respectively). Finally, reads coverage and number of VIS loci in mixed and diluted samples were comparable between LM-NEO-PCR alone and LM-NEO-PCR combined with capture methods (Fig. 4E,F). No significant differences in reads coverage and number detected VIS loci were observed when comparing PCR alone with each capture method or between both capture methods. Summarizing, our developed VIS-NGS protocol seems to specifically enrich loci with high reads coverage when compared to negative controls. The protocol performance was further investigated for its specificity by examining VIS loci in DNA from pure clones and from dilution and mixtures of clones with reported VIS sites.

### Detection of reported and new VIS loci in pure VIS clone and parental samples by LM-NEO-PCR

**Fig. 5:**
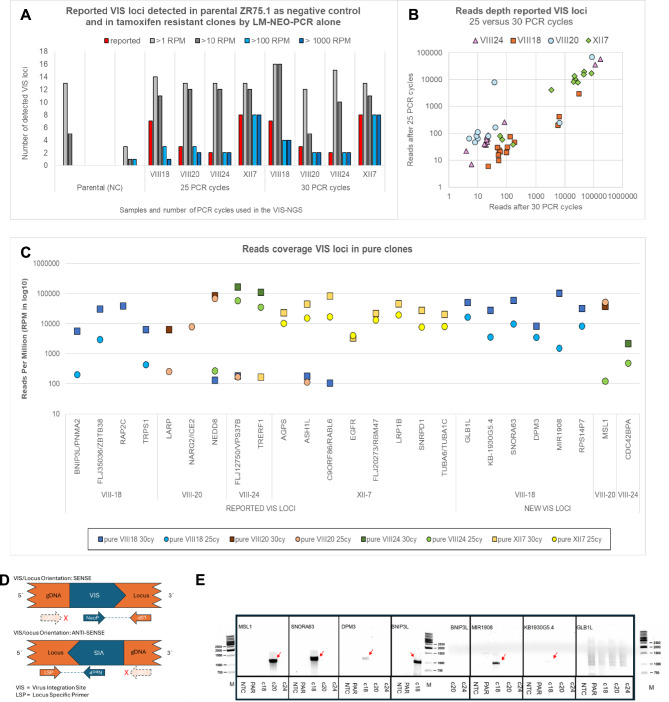
Detection of reported and new VIS loci in single pure clones and by VIS-NGS LM-NEO-PCR alone. Detection of reported and new VIS loci in single pure clones after LM-NEO-PCR. The number and read depth coverage of the previously reported VIS loci in the single pure clones are presented in (**A**) and (**B**) for both 25 and 30 PCR amplification cycles. (**A**) The number of loci detected per clone at four thresholds for read depth coverage. It shows that > 1 and > 10 RPM calling (grey bars) result in more loci in each clone than reported (red bar), irrespective 25 or 30 PCR cycles, but also in off-target calling in negative controls (parental). The thresholds > 100 and > 1000 RPM (blue bars) result in numbers of VIS loci called (most) like the number reported. (**B**) Read depth coverage per detected locus after 25 and 30 PCR cycles. It shows two separate groups of loci, one group with low read depth coverage (< 100 RPM) and the other group of loci with high read depth coverage (> 1000 RPM). (**C**) Presents the read depth coverage for each reported and new VIS locus, showing that reported VIS loci were correctly called at > 1000 RPM, while a-specific calling occurs around 100 RPM in other clones than reported. Moreover, it shows that as expected 30 PCR cycles (squares) resulted generally in higher read depth coverage than 25 PCR cycles (circles). All novel VIS loci were found based on the > 1000 RPM calling threshold. Only *MSL1* was detected in clone VIII-20, but also a-specifically at lower read depth coverage in clone VIII-24. (**D**) Illustrates the PCR design with the universal neomycin primer (neoP) which can act as forward and reverse primer depending on the VIS-locus orientation. Forward and reverse locus-specific primers (LSPs) were developed but only one LSP together with neoP resulted in PCR amplification products. The locus-specific PCR and gel electrophoresis analyses were performed for one reported locus (*BNIP3L*) and six novel VIS loci identified by VIS-NGS in clones VIII-18 (*DPM3, MIR1908, KB1930G5.4, SNORA63, GLB1L*) and VIII-20 (*MSL1*) (**E**). PCR amplicons were only obtained when the neomycin primer and locus-specific primer had an opposite orientation. The expected > 1000 bp PCR amplicons were seen for all except *GLB1L* in only the correct clones (indicated by red arrows). No amplicons were observed in the negative template control (NTC) nor in parental without VIS (PAR), nor in clones lacking the VIS locus.

The DNA samples from pure clones with known VIS loci were evaluated using the LM-NEO-PCR protocol (25 or 30 PCR cycles) alone followed by NGS. To show specificity, we employed DNA from four previously generated tamoxifen resistant ZR75.1 subclones: subclone VIII-18, VIII-20, VIII-24, and XII-7 (4) which carried 20 VIS loci (7, 3, 2 and 8, respectively) in their genome. In subclone VIII-18 one VIS locus was not detected (*LOC391679*) and two VIS loci failed quality control (*FSTL5 and SRD5AP1,* detected only once and with less than 100 reads, see methods). The remaining seventeen VIS loci (89%) were all found in replicates including the corresponding pure tamoxifen resistant clone but also (non-specifically) at lower read depth coverage in pure samples from other clones (Fig. 5A, Supplemental Table S3). Moreover, the reported VIS loci were seen in parental samples, acting as negative controls (NC, Fig. 5A), but were mainly detected at low read depth coverage (< 100 RPMs); only one VIS locus (*FLJ12750/VPS37A/VPS37B*) was detected with > 100 RPMs. All this demonstrates the high specificity of the VIS-NGS protocol for enrichment of VIS loci in retroviral infected cells. Comparing the number of PCR cycles performed (25 versus 30) showed as expected higher reads coverage for almost all reported VIS loci with more amplification cycles (Fig. 5B,C). Two loci (*RAP2C, NARG2/ICE2*) were called only once, while all other loci were detected in both PCR runs (Fig. 5C). Fifteen VIS loci (88%) were detected with > 100 RPMs coverage with both PCR amplification cycles, and again at high reads coverage (> 1000 RPMs) with 30 PCR cycles while only 13 VIS loci (76%) were detected with 25 cycles (Fig. 5C). None of reported VIS loci were observed in other clones when applying a > 1000 RPMs threshold, while at > 100RPMs two loci after 25 PCR cycles and five loci after 30 PCR cycles were detected in incorrect clones, respectively. Based on > 100 RPMs findings, we assessed the diagnostic performance of the VIS-NGS protocol. This resulted in a sensitivity of 89% and 76%, a specificity of 94% and 92%, and an accuracy of 91% and 82% for 25 and 30 PCR cycles, respectively. Interestingly, eight not yet reported VIS loci were found in pure clones, almost all with read coverage higher than 1000 RPMs irrespective the number of PCR cycles (Fig. 5C). Six loci were discovered in clone VIII-18 (*GLB1L, KB-1930G5.4, SNORA63, DPM3, MIR1908,* and *RPS14P7*), one locus in clone VIII-20 (*MSL1*), and one in clone VIII-24 (*CDC42BPA*). Summarizing, the LM-NEO-PCR was able to find 85% of the reported VIS loci in the correct clones, and when applying 30 PCR amplification cycles all seventeen but one reported and eight potentially novel VIS-loci were detected at a coverage of more 1000 RPMs, a threshold excluding false positive findings in other clones and negative controls.

### Detection of reported and new VIS loci in composed multiplex samples and comparison of LM-NEO-PCR alone or combined with additional capture methods

**Fig. 6:**
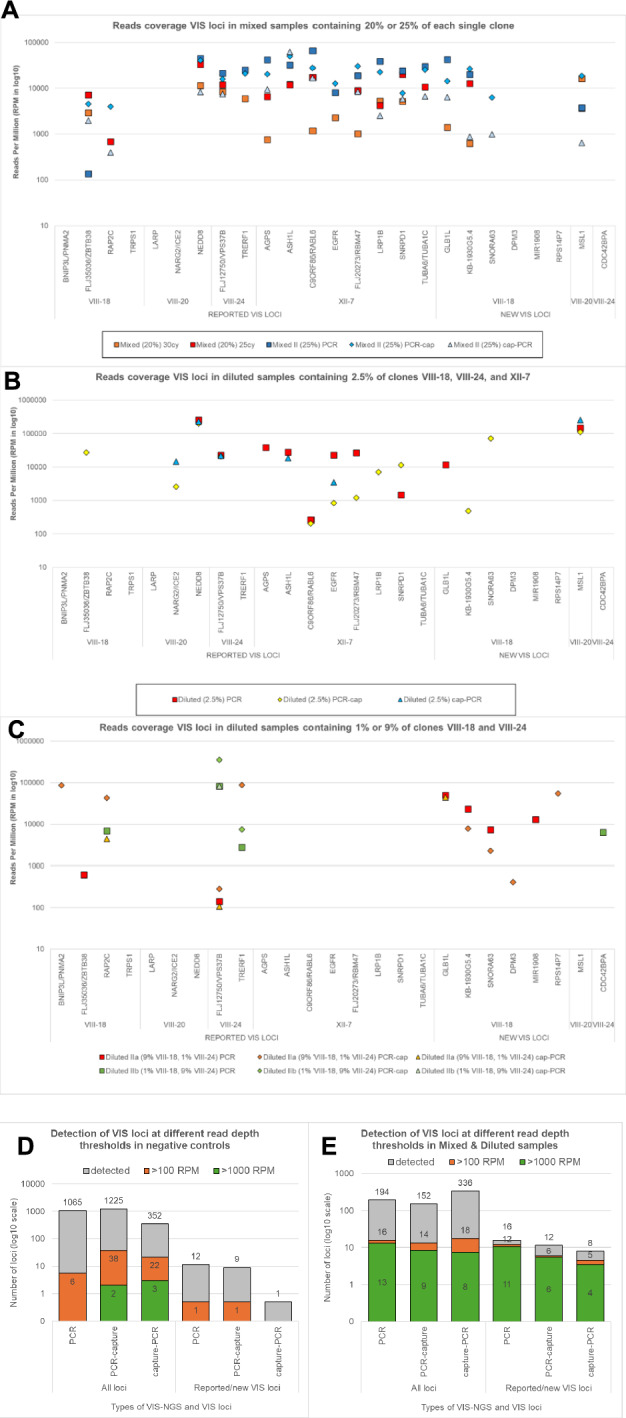
Detection of reported and new VIS loci in mixed and diluted samples evaluated by LM-NEO-PCR alone or combined with NEO-capture. Evaluation of the VIS-NGS performance in composed mixed and diluted samples. The LM-NEO-PCR alone or combined with NEO-capture before (neo-PCR) or after (PCR-neo) were used to detect VIS loci in composed DNA samples with 2 or 4 pure clones mixed and diluted up to a clone proportion of 1%. (**A**) The VIS loci detected in mixed samples with 20% and 25% of each clone. Seventeen of the 25 reported and new loci identified in single clones were detected in these mixed samples. (**B**) and (**C**) Present the VIS loci detected in diluted samples containing either a proportion of 2.5% for clones VIII-18, VIII-24, and XI-7 (**B**) and of 9% or 1% for clones VIII-18 and VIII-24 (**C**). Half of the reported VIS loci were detected in samples containing a proportion of 1% for one clone. (**D**) and (**E**) show the number of all and reported/new loci detected in negative controls (**D**) and in mixed and diluted samples (**E**) after LM-NEO-PCR alone, PCR-capture, of capture-PCR. The numbers are presented for three thresholds of read depth coverages (> 1 RPM, > 100 RPM, > 1000 RPM).

Specimens were mixed (2 or 4 clones mixed) and diluted (1 in 4 till 1 in 100) with the aim to mimic samples used in screens having VIS randomly integrated but also with the aim to gain insight in specificity during declining sensitivity. The composed samples were evaluated with LM-NEO-PCR alone or combined with one of the two neomycin capture methods mentioned above (Fig. 2). The purpose was to find the best method for specific and sensitive VIS-locus enrichment in mixed samples and diluted samples with low clone proportions. Therefore, the calling and read depth coverage of the seventeen reported and eight new VIS loci found in the pure clones was first determined in mixed samples containing 25% or 20% of each pure clone. In total, thirteen reported (76%) and four novel (50%) VIS loci were detected in the mixed samples by one or more methods (Fig. 6A, Supplemental Table S3). The PCR-capture method detected the highest percentage of VIS loci and at significant higher median read coverage (17 loci (68%) detected at median 20,292 RPMs) when compared to the capture-PCR method (15 loci (60%) detected at 6417 RPMs, *p* = 0.032) and LM-NEO-PCR alone (15 loci (60%) detected at 10,707 RPMs, *p* = 0.025).

Analyses of the six reported VIS loci from clones VIII-18 and VIII-24, which were potentially measurable in all multiplexed and diluted samples, showed that only *RAP2C*, *VPS37A*, and *TRERF1* were detected in the composed sample at a proportion of 1% of the respective clone (Fig. 6C). Moreover, *VPS37A* was detected by all three methods in all composed samples, while *RAP2C* and *TRERF1* were detected in the 1% proportion sample by PCR alone and PCR-capture, respectively. Both latter loci were also detected predominantly by PCR-capture in composed samples having the relevant clone present at a higher proportion (9% and 25%) but not at 2.5% proportion (Supplemental Table S3). Extending the evaluation of the diluted sample having a relevant clone for clone XII-7 at a 2.5% proportion showed that PCR alone and PCR-capture detected six and five of its eight reported loci (> 62.5%), respectively, while capture-PCR called only two loci (25%) (Fig. 6B).

The four novel VIS loci detected in the single pure clone and in the mixed clones′ samples (Fig. 6A, *GLB1L, KB-1930G5.4, SNORA63 (*Clone VIII-18)*; MSL1 (*Clone VIII-20)) were also evaluated in the diluted samples. The VIS locus *MSL1* for clone VIII-20 could not be verified in diluted samples since proportions below 20% for this clone were not assessed. The three VIS loci from VIII-18 were found in diluted samples with a 9% proportion of this clone by both PCR alone and capture and at a 2.5% proportion by PCR alone (*GBL1L*) or by PCR-capture (*KB-190G5.4, SNORA63*) but none by capture-PCR. These loci were not detected at a 1% proportion of clone VIII-18. The remaining novel VIS loci from clone VIII-18 (*DPM3, MIR1908, RPS14P7)* and clone VIII-24 (*CDC42BPA*) were not observed in mixed samples but were detected in diluted samples with a 9% but not a 2.5% nor 1% proportion of clone VIII-18 or VIII-24 by PCR alone (*MIR1908, CDC42BPA*) or by PCR-capture (*DPM3, RPS14P7*) (Fig. 6C).

Comparing the number of all loci and reported/new VIS loci identified by PCR alone and by capture methods in negative controls and combined in mixed and diluted samples showed no significant differences between methods irrespective read depth coverage (*p* > 0.37) (Fig. 6D,E). Applying a high read depth coverage threshold (> 1000 RPM) for locus calling resulted in median numbers for all loci and reported/new VIS loci of 13 and 11 for PCR alone, 9 and 6 for PCR-capture, and 8 and 4 for capture-PCR, respectively (Fig. 6E). Next, the performance of each method was determined based on the numbers of reported/new loci versus all loci, with specificity and sensitivity (using the total of 17 VIS loci identified in the mixed samples (Fig. 6A: 13 reported loci & 4 new loci) as reference) as follows: for PCR alone a specificity of 85% (11/13 loci) and a sensitivity of 65% (11/17 loci) was achieved, for PCR-capture 67% (6/9 loci) and 35% (6/17 loci), and for capture-PCR 50% (4/8 loci) and 24% (4/17 loci) (Fig. 6E). For diluted samples, however, lower read coverage per locus can be expected since the VIS locus itself is less abundant, therefore, a less stringent threshold for read depth coverage might perform better. When applying a threshold of > 100 RPM, specificity and sensitivity are for PCR alone 75% (12/16 loci) and a 71% (12/17 loci), for PCR-capture 43% (6/14 loci) and 35% (6/17 loci), and for capture-PCR 28% (5/18 loci) and 29% (5/17 loci).

Summarizing, the LM-NEO-PCR alone identified overall slightly more VIS loci than PCR-capture or capture-PCR and detected at least half of the reported VIS loci at 1% and 2.5% clone proportions.

### Independent confirmation of novel VIS loci

One reported VIS locus (*BNIP3L*) and six novel VIS loci identified in clones VIII-18 and -20 (*DPM3, MIR1908, KB1930G5.4, SNORA63, GLB1L* (clone VIII-18); and *MSL1* (clone VIII-20) were verified by PCR and agarose gel electrophoresis analyses. Figure 5E shows the PCR products obtained in negative controls (NTC, PAR) and in clones VIII-18, -20, and -24. The expected > 1000 bp PCR amplicons were seen for all except *GLB1L* in the correct clones. These fragments were only obtained for the locus-specific primers with the same orientation of the VIS, while not observed in the negative controls, nor in clones lacking the specific locus, nor obtained by locus-specific primers with the opposite orientation of the VIS (data not shown).

## Discussion

Retroviruses insert their genomic material at different sites in the host genome which will change the chromosome architecture and may affect downstream gene expression at the site of insertion. Exploiting this peculiar property, we set out to develop a protocol for rapidly identifying genes having a VIS nearby putatively driving resistance to endocrine therapy. Using tamoxifen-resistant breast cancer cell line clones previously derived from retrovirally infected parental ZR-75-1 cells^3^, we provide evidence that we have developed an easy and efficient LM-NEO-PCR/NGS-based protocol associated to a genome walker adapter to enrich and sequence viral and host genome junctions. Also, a bioinformatic pipeline used to analyze the NGS data and able to find both VISs genomic coordinates and associated genes is described. Finally, especially the LM-NEO-PCR alone and PCR-Capture methods were able to detect reported VIS-loci in mixed specimens with clones present at low frequency.

Here, we developed a restriction enzyme-independent LM-PCR protocol associated to genome walker adapter system for virus-genome junction identification by NGS analysis. Our approach appears to be an easy method applicable to any retroviral vector having a unique site up-stream of the 3′LTR region. Employing previously characterized ZR75.1 infected clones, we evaluated the specificity and sensitivity of LM-PCR associated to genome walking of three methods to enrich the virus sequence. To this end we used the neomycin region as unique site followed by selective amplification of the 3′LTR-genome junction then subjected enriched material to NGS. This method was able to detect almost all reported VIS-loci correctly in single pure clones. Alternatively, the neomycin region was also captured before or after PCR and followed by NGS. All three methods were able to find reported VIS-loci after NGS in VIS clone mixtures at low clone fractions but each at slightly different sensitivity and specificity. Considering that both LM-NEO-PCR and PCR-Capture were more effective than Capture-PCR, confirms the results obtained by NEO directed PCR as a more efficient approach. Indeed, PCR-Capture works using direct NEO-PCR followed by NEO-capture when compared to capture-PCR, which performed the capture step before the NEO-PCR, suggesting that performing PCR on the neomycin region as first step results in a more efficient way of detecting target loci having a VIS. These data support the hypothesis that a capture step, performed before virus enrichment, requires more abundant VIS loci to correctly pull out the virus sequence from a mixed population.

Finally, the observation that (almost) none of the reported VIS loci have been identified at higher read depth coverages (> 100 RPMs and > 1000 RPMs) in no-template negative control (ZR75.1 parental cells) indicates that, our system selectivity recognized the VIS allowing to apply cut-offs, expressed as RPM, to minimize the noise findings derived from both PCR, capture and NGS experimental conditions. Moreover, five of the six novel VIS loci identified by VIS-NGS at > 100 RPMs were confirmed by independent PCR and gel electrophoresis analyses, showing that the VIS-NGS enables the detection of new VIS loci not yet reported by conventional methods in our retroviral clones.

In summary, in this study our method was applied to find the gene locus involved in the retroviral integration site on endocrine therapy resistant BC cells. Its flexibility makes it possible to be used in any cellular model to study multiple (dominant and minor) genetic alterations effects in retroviral screens for therapy resistance.

## Supplementary Information

Supplementary Information.

## Data Availability

The VIS-NGS fastq-files have been deposited at the NIH Sequence Repository Archive (SRA) and are accessible within BioProject PRJNA1210462 (SUB15007969). Furthermore, data generated in this study and supplementary data files are available upon request by emailing the corresponding author (m.p.h.m.jansen@erasmusmc.nl).
