# Supplementary material for: Novel protocol for mapping virus integration sites in genes involved in therapy resistance
Source: Sci Rep. 2025 Jul 1;15:21841. doi: 10.1038/s41598-025-05160-4 (PMC12215797; doi:10.1038/s41598-025-05160-4)

**Supplemental Tables and Figure & Supplemental Methods file**

**Supplemental table S1 Linkers, primers & probe details used in the different steps of the LM-NEO-PCR-based VIS-NGS protocol.**

**Supplemental Table S2 Sample details and summary of LM-NEO-PCR VIS-NGS results**

**Supplemental Table S3 - Reported and novel VIS loci and detection in pure clone DNA samples and in composed mixed and diluted DNA samples**

**Supplemental Table S4 – PCR Primers and thermal cycling conditions used for the independent validation of VIS loci by PCR and gel electrophoresis analysis.**

*Thermal cycling conditions (bio-rad T100 thermal cycler)*

**Supplemental Figure S1** – Workflow of capture approaches to enrich VIS holding gDNA.


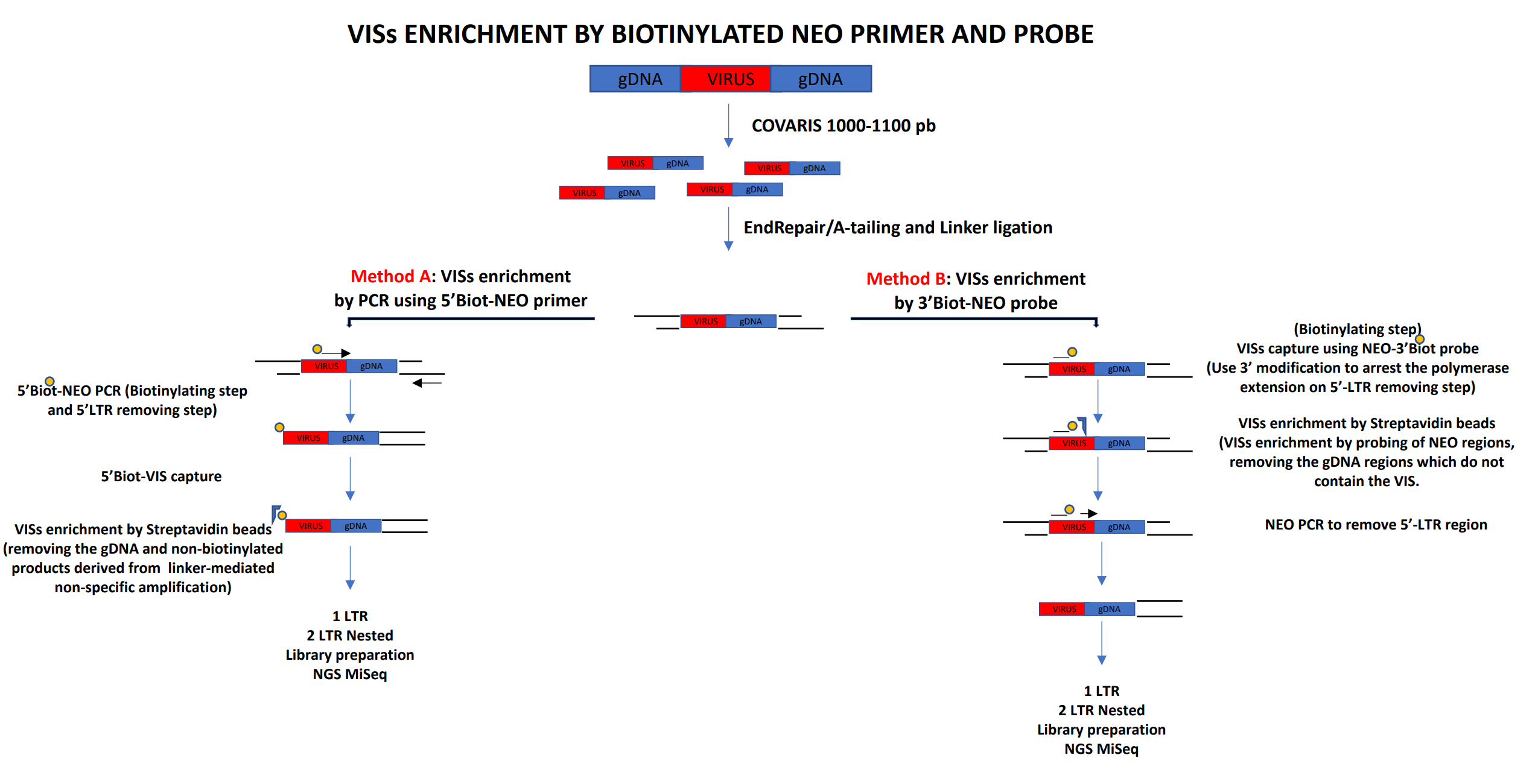


Two different capture methods were applied to obtain virus enriched samples. Method A used a NEO biotinylated primer followed by capture approach, while method B performed virus enrichment by capture using a biotinylated NEO probe followed by NEO-PCR. Then, both virus enriched samples have been subjected to first and nested LTR PCR.

**Supplemental Figure S2** **– Gel electrophoresis analyses of PCR fragments.**


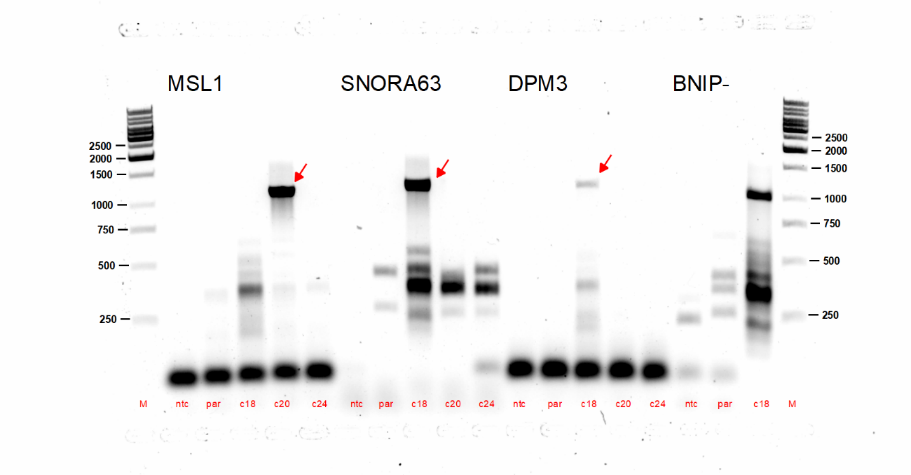


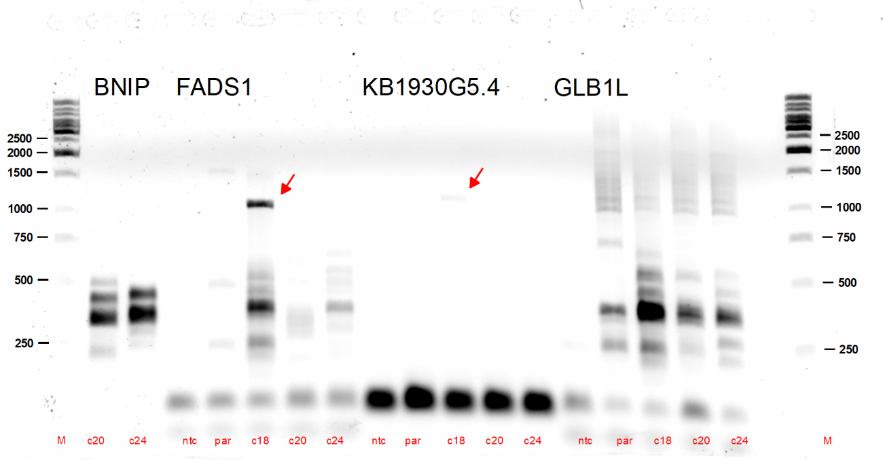


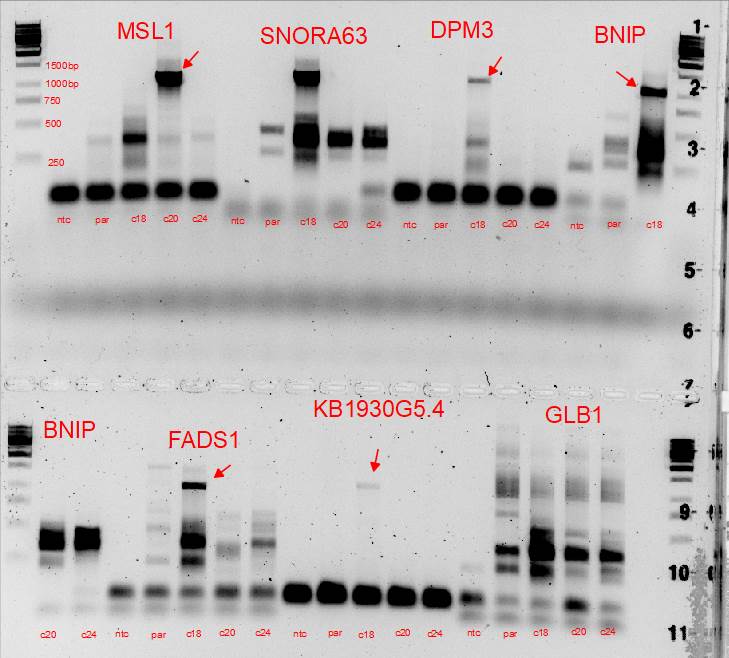


Original gel electrophoresis images with and without contrast adjustment obtained by chemidoc recording of PCR fragments after independent confirmation of novel VIS loci by PCR presented in detail in figure 5E.

***Supplemental File - Method details***

*Methods*

*In this section we describe step-by-step the methods used to generate and sequence a VIS library. Starting from gDNA sheared by sonication we used a “Genome walker adapter system” able to selectively amplify 3’LTR downstream regions using the neomycin gene as unique and not repeated virus sequence.*

*gDNA shearing by COVARIS sonication.*

gDNA, derived from different retrovirally infected ZR-75-1 tamoxifen-resistant BC cell lines, was sheared by sonication employing Covaris sonicator to obtain fragments size of ~1.000-1.100 bp as average according to manufacturer’s protocol for S220 tube (Covaris, https://www.covaris.com/protocols). To remove shorter gDNA fragments, we performed left side size selection (this method removes DNA fragments that are smaller than the targeted size) employing AMPure XP using 0.7x DNA/beads ratio as follows. 0.7x AMPure XP was added to the supernatant containing fragmented gDNA and incubated for 5 minutes at room temperature (RT), then placed on magnetic stand for 3 minutes. At this time the supernatant was discarded, and the beads washed two times with 70% ethanol. The beads were dried for 5 minutes to completely remove the ethanol and gDNA eluted by Low TE. Qubit fluorometer was used to quantify the purified gDNA and 1ng analyzed by Bioanalyzer HS DNA chip to evaluate the correct distribution of DNA fragments.

*End-repair and 3’A-Tailing and linker ligation.*

Sticky-end DNA breaking was end-repaired and 3’A-Tailed using NEBNext Ultra II End Repair/dA-Tailing Module (NEB). Briefly, gDNA were treated with NEBNext Ultra II End Prep Reac Mix and End Prep Enz Mix in a total volume of 60 mL for 30 minutes at 20 °C followed by 30 minutes at 65 °C.

*Annealing of asymmetric linker and ligation*

An asymmetric linker (Table S4) containing 3’dT-overhang compatible with 3’dA-overhang of the repaired gDNA was prepared as follows. Both shorter and longer linkers have been annealed in TrisEDTA1x (TE1x) buffer supplemented with 50mM of NaCl in a total volume of 20µL using 15µM as final concentration for each. In a thermocycler both linkers have been denatured at 90 °C for 2 minutes, then annealed by slowly cooling to 15 °C in steps of 1 °C per minute. At this time repaired gDNA and annealed asymmetric linker were mixed, and ligation performed at 12 °C overnight employing NEBNext Ultra II Ligation Module (NEB). AMPure XP was used for purification using 1.2x as DNA/beads ratio.

 ***Table S4***

*Neomycin-PCR for virus sequence enrichment (method 0)*

Linked DNA was directly used for NEO-PCR according to the following protocol. GoTaq G2 Flexi DNA polymerase (Promega) was used according to the manufacture’s protocol for all PCR reactions Neo-PCR was performed using NEO-specific primer and linker-specific primer (Table S5) according to the tables S6 and S7.

**Table S****5**

**Table S6 *(NEO PCR set-up)* Table S7 *(NEO PCR Thermal Cycling Conditions)***

After NEO-PCR, the amplified product was cleaned using AMPure XP beads by a 1.2x DNA/beads ratio.

*VISs enrichment by PCR-Capture using 5’Biot-NEO primer (method A)*

To perform VISs enrichment by PCR followed by capture approach we used 5’-Biot_NEO primer as biotinylating step to capture and enrich the virus sequence as follows. Linker specific and 5’Biot-NEO primers (Table S8) were used to perform first NEO-PCR by reagents and cycling conditions reported in tables S6-S7.

**Table S8**

The biotinylated product was purified using AMPure XP by 1.2x DNA/Beads ratio. Then dynabeads M-270 Streptavidine was used according to the manufacture’s protocol (Invitrogen) to capture the biotynilated PCR products. At this time the first and nested LTR-PCR were performed to selectively amplify the LTR-genome junction.

*VISs enrichment by Capture-PCR using 3’Biot-NEO primer (method B)*

With this method we performed virus enrichment using a biotinylated primer which selectively isolates the virus sequence that next was subjected to NEO-PCR. To this end we used a NEO_3’Biot probe (table S9) on linked DNA which was subjected to NEO enrichment by dynabeads M-270 according to manufacturer’s protocol (Invitrogen). Then NEO-PCR was performed using Neo-specific primer(2) (Table S9) according to the experimental conditions reported in tables S6-S7.

**Table S9**

*3’LTR-PCR and 3’LTR Nested PCR*

The virus enriched samples obtained by LM-NEO-PCR or NEO-capture were subjected to selective 3’-LTR-genome junction amplification by the following protocol.

3’LTR PCR was performed using linker-specific primers (table S5) and 3’LTR-specific primers reported in table S10.

**Table S10**

The PCR experimental conditions are reported in tables S8-S9.

**Table S11- *3’LTR PCR set-up* Table S12 - *3’LTR PCR Thermal Cycling Conditions***

After PCR, the amplified product was cleaned using AMPure XP beads by 1.8x DNA/beads ratio and used to perform 3’LTR nested PCR.

The 3’LTR Nested PCR was performed using 3’LTR-specific primer (Nested, Table S10) and linker-specific primer (Table S5) according to the tables S11-S14.

**Table S13 - *3’LTR PCR set-up* Table S14- *3’LTR PCR Thermal Cycling Conditions***

After nested PCR, the 3’LTR-genome junction amplified product was purified applying right selection to remove larger fragments. 0.7x AMPure XP beads have been added to the PCR product and incubate for 5 minutes at RT, then placed on a magnetic stand for 3 minutes. The supernatant was then transferred in a new tube and the beads discarded. The solution was mixed with 1.2x (according to initial volume of the PCR product) of AMPure XP and incubated for 5 minutes to room temperature and placed for 3 minutes on magnetic stand. At this time the supernatant was removed, and the beads washed two times with 70% ethanol. The beads were dried for 5 minutes, and PCR product eluted in Low TE.

*TruSeq DNA PCR-free for NGS library preparation*

NGS library preparation was generated using TruSeq DNA PCR-free from Illumina. 3’LTR-genome junction PCR product was end-repaired in RSB buffer and ERP mix at 30 °C for 30 minutes and purified by SPB using 1.6x as DNA/beads ratio as follows. SPB were mixed with PCR product and incubated for 5 minutes at r.t then placed on magnetic stand for 3 minutes. The supernatant was discarded and SPB washed two times by 80% ethanol solution. SPB were dried and cleaned PCR product collected in RSB. The repaired DNA was A-Tailed using ATL mix at 37 °C for 30 minutes followed by 70 °C for 5 minutes. Nextera Illumina i5 and i7 dual index were then ligated as adapter for sequencing using LIG2 mix at 30 °C for 10 minutes. Each library was purified using left - right selection to collect amplicons having 300-500 pb. For right selection 0.61x of SPB were added in DNA supernatant and incubated for 5 minutes at RT, then placed in magnetic stand for 3 minutes. The supernatant was transferred in a new tube and beads discarded. Left selection was performed adding 0.2x of SPB to the supernatant and incubate for 5 minutes at RT. At this time the supernatant was discarded and SPB washed two times with 80% of ethanol. DNA was eluted in LowTE and quantified by Qubit fluorometer, and 1 ng analyzed by Bionalyzer HS DNA chip to evaluate the fragment size distribution. For library quantification NEBNext Library Quant Kit for Illumina was used and each library pooled at final concentration of 2nM for each and sequencing performed using Mi-Seq Illumina platform.

**Processing of VIS Sequencing data**

The following pipeline was used to process paired-end FASTQ files for virus integration site (VIS) analysis (Supplemental Figure S3). First the CUTADAPT (version 3.7) tool was used to trim the 3’ LTR adapter (GGGTCTCCTCTGAGTGATTGACTACCCACGACGGGGGTCTTTCA) and the 5’ linker (GTAATACGACTCACTATAGGGCCTCCGCTTAAGGGACT) from raw reads, using an error rate 0.25, discarding untrimmed reads and a minimum read length of 36bp.

Next, “BWA (version 0.7.17-r1188) mem” with default settings was used to map the trimmed FASTQ files to the hg38 genome (hg38.AnalysisSet). SAMTOOLS (version 1.9) was then used for converting the resulting alignment files (SAM) to BAM, sorting, indexing, and removing reads with mapping quality values below 20.

Coverage of each Virus Intergration Site was computed utilizing “BEDTOOLS (version 2.30) genomecov” and covered regions within a 300bp (approximate insert size of library) window were merged with “BEDTOOLS merge” and considered a single VIS. Using “BEDTOOLS intersect”, each VIS that overlapped with gene coordinates was annotated with the corresponding gene names from NCBI Refseq, which were obtained via the “UCSC TableBrowser” tool. Additionally, “BEDTOOLS closest” was used to find the gene nearest to each VIS and computing the distance to the VIS based on the NCBI Refseq gene data. For further filtering and VIS calling reads were normalized per million (RPM) and a threshold of >100 RPM was considered.

Supplemental Figure S3. Overview of VIS sequencing data processing


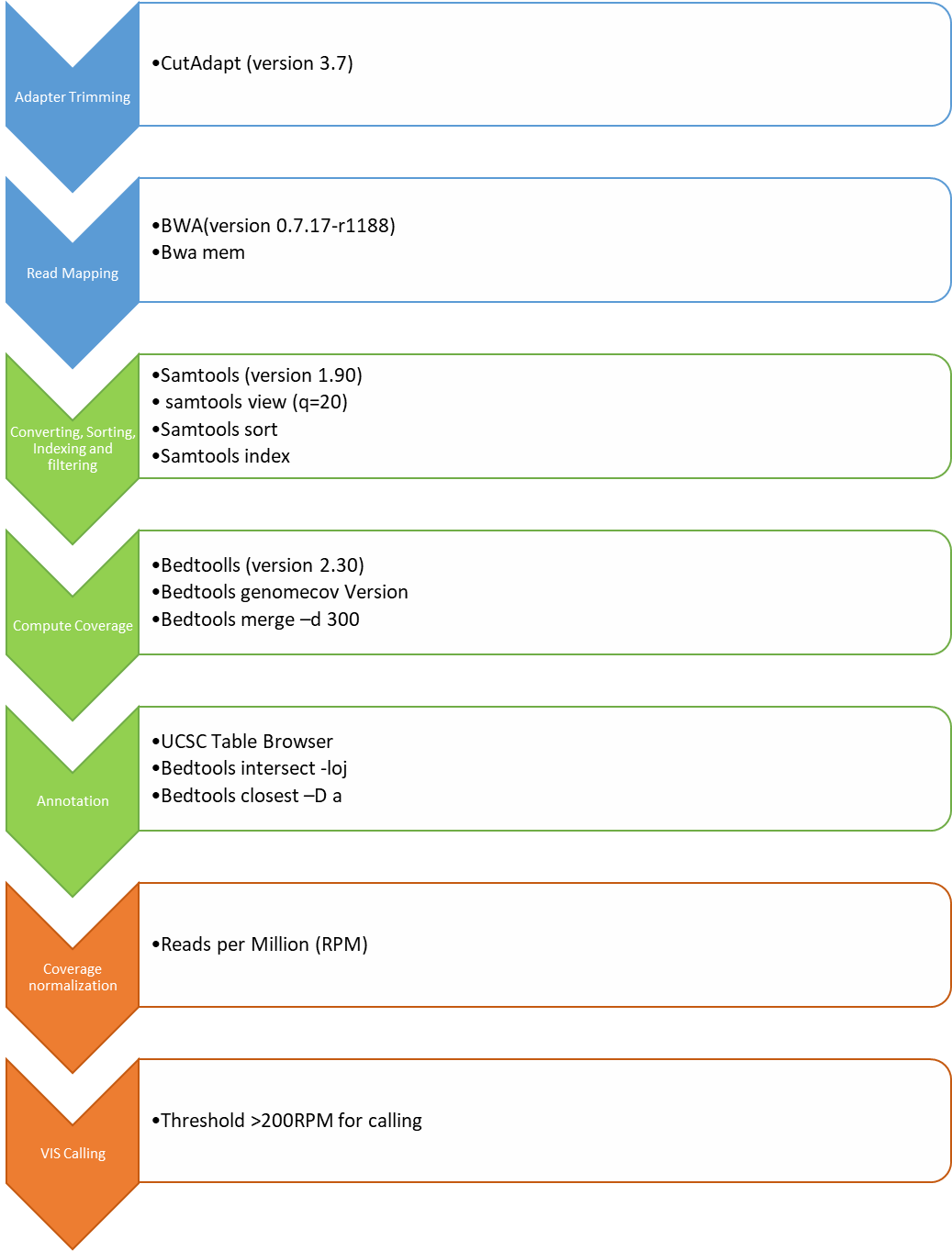

Supplement: Supplementary file 1 — Supplementary Information. [file 41598_2025_5160_MOESM1_ESM.docx]
